# New Thiazole Acetic Acid Derivatives: A Study to Screen Cardiovascular Activity Using Isolated Rat Hearts and Blood Vessels

**DOI:** 10.3390/molecules27196138

**Published:** 2022-09-20

**Authors:** P. Raghunatha, Mohammed Naseeruddin Inamdar, Syed Mohammed Basheeruddin Asdaq, Mansour Almuqbil, Abdullah R. Alzahrani, Saleh I. Alaqel, Mehnaz Kamal, Firas Hamdan Alsubaie, Walaa F. Alsanie, Abdulhakeem S. Alamri, Syed Imam Rabbani, Mahesh Attimarad, S. Mohan, Majid Alhomrani

**Affiliations:** 1 Department of Pharmacology, Al-Ameen College of Pharmacy, Bangalore 560027, India; 2 Department of Pharmacology, East West College of Pharmacy, Bangalore 560091, India; 3 Department of Pharmacy Practice, College of Pharmacy, AlMaarefa University, Dariyah, Riyadh 13713, Saudi Arabia; 4 Department of Clinical Pharmacy, College of Pharmacy, King Saud University, Riyadh 11451, Saudi Arabia; 5 Department of Pharmacology and Toxicology, Faculty of Medicine, Umm Al-Qura University, Al-Abidiyah, P.O. Box 13578, Makkah 21955, Saudi Arabia; 6 Department of Pharmaceutical Chemistry, Faculty of Pharmacy, Northern Border University, Rafha 91911, Saudi Arabia; 7 Department of Pharmaceutical Chemistry, College of Pharmacy, Prince Sattam Bin Abdulaziz University, Al-Kharj 11942, Saudi Arabia; 8 Medical Sale Representative, Jamjoom Pharmaceutical Company, Riyadh 12211, Saudi Arabia; 9 Department of Clinical Laboratory Sciences, The Faculty of Applied Medical Sciences, Taif University, Taif 21944, Saudi Arabia; 10 Centre of Biomedical Sciences Research (CBSR), Deanship of Scientific Research, Taif University, Taif 21944, Saudi Arabia; 11 Department of Pharmacology and Toxicology, College of Pharmacy, Qassim University, Buraydah 51452, Saudi Arabia; 12 Department of Pharmaceutical Sciences, College of Clinical Pharmacy, King Faisal University, Al-Ahsa 31982, Saudi Arabia; 13 Faculty of Pharmaceutical Sciences, PES University, Bengaluru 560085, India

**Keywords:** isolated heart, isolated blood vessel, thiazole acetic acid derivatives, Langendorff apparatus, cardiovascular activity

## Abstract

Cardiovascular diseases are one of the major causes of mortalities worldwide. In the present research, new synthetic derivatives of thiazole were studied using isolated hearts and blood vessels of rats. The heart and thoracic aorta were tested with six new synthesized thiazole acetic acid derivatives (SMVA-10, SMVA-35, SMVA-40, SMVA-41, SMVA-42 and SMVA-60), and the data obtained were statistically analyzed and compared. Isolated rat hearts were used to record the changes in developed tension and heart rate, while thoracic aortas were used to measure the contractile response, before and after treatments. Analysis of the results indicated a significant (*p* < 0.01) increase in developed tension with the addition of SMVA-35, SMVA-40, SMVA-41 and SMVA-42, which was augmented in the presence of adrenaline without affecting the heart rate. On the other hand, acetylcholine significantly decreased the developed tension, which was significantly reversed (*p* < 0.01) in the presence of compounds (SMVA-35 and SMVA-60). However, in the presence of SMVA-35 and SMVA-40, acetylcholine-induced bradycardia was significantly (*p* < 0.01) reduced. Furthermore, only SMVA-42 induced a dose-dependent contractile response in the isolated blood vessel, which was abolished in the presence of prazosin. Therefore, it can be concluded that some of the new synthesized thiazole derivatives exhibited promising results by raising the developed tension without changing the heart rate or blood vessel function, which could be helpful in failing heart conditions. However, more research is required to fully comprehend the function, mechanism and effectiveness of the compounds.

## 1. Introduction

The search for a novel therapeutic intervention is an evergreen segment in the field of medical research. Drug screening in recent times has not only become high-tech but also highly intense and advanced [1]. Due to industrialization, the major causes of death and disability in more advanced societies have now shifted from the predominance of nutritional deficiencies and infectious diseases to degenerative ailments such as cardiovascular diseases (CVDs), cancer, and diabetes. This shift has been named “the epidemiologic transition” [2].

One of the major reasons for higher incidences of CVD in the population is the association with increasing levels of dyslipidemia, high blood pressure and diabetes [1,2]. Among the ailments, CVD is considered to be one of the leading causes of mortality in developing countries [3]. Different medical interventions that include several classes of drugs have been approved for treating cardiovascular diseases [4].

The medications for metabolic disorders are mostly prescribed for a longer period of time and could lead to several complications. The long-term use of beta-blockers has been linked to claudication in patients with peripheral vascular disease and bronchoconstriction in patients with chronic obstructive pulmonary disease [5]. The chronic use of angiotensin-converting enzyme inhibitors has also been linked to kidney disease, while statins used to lower cholesterol can cause liver damage, renal insufficiency, and myalgias [6,7]. Even though many medications are available, the search for safe and effective therapeutic interventions to treat cardiovascular diseases is a major global healthcare challenge [4,5].

As reported in the literature, heterocyclic compounds occupy a unique place in medicinal chemistry. These compounds exhibit large-scale biological activities due to their ability to interact with different receptors. Furthermore, these agents can be easily synthesized or can be found as active ingredients in several naturally derived medicinal substances [8].

Different heteroatoms containing compounds have been reported to exhibit pharmacological activities such as anticancer, antiviral, anti-inflammatory, antidiabetic, antihypertensive, antitubercular and anticonvulsant effects [9]. Thiazole-containing heterocyclic compounds were also found to possess numerous biological activities [10]. The agents were found to be effective even in cancer and HIV in addition to the already reported activities [10,11,12]. The search for newer activities for this class of compounds is intensifying as a growing number of activities are reported [13,14]. Additionally, substitutions such as O-, N- and S- were found to play an important role in the modification of both the pharmacokinetic and pharmacodynamic properties of the heterocyclic compounds [15]. Hence, the present study was planned to screen the new synthesized derivatives of thiazole acetic acid for cardiovascular effects using isolated hearts and aortas in experimental rats.

## 2. Results

### 2.1. Effects of New Synthetic Derivatives of Thiazole Acetic Acid on Developed Tension (gm) in Isolated Rat Heart

Different concentrations of new synthetic derivatives of thiazole acetic acid (SMVA-10, 35, 40, 41, 42 and 60) were tested to evaluate their effect on the developed tension in an isolated rat heart. When compared to the control, SMVA-10 induced a significant (*p* < 0.05) increase in developed tension at the lower dose (1 µM). SMVA-35 significantly (*p *< 0.05) increased the developed tension at 10 nM, 100 nM, 1 µM and 100 µM compared to control. SMVA-40 resulted in a significant (*p *< 0.05) increase in developed tension at all the tested doses except 10 µM. On the other hand, both SMVA-41 and SMVA-42 produced a pronounced increase (*p *< 0.001) in developed tension at all the tested doses. However, SMVA-60 produced enhanced developed tension (*p *< 0.05) only at lower doses, while the higher doses (1, 100 and 300 µM) did not produce a significant increase compared to the control group (Table 1).

### 2.2. Effects of New Synthetic Derivatives of Thiazole Acetic Acid on Heart Rate in Isolated Rat Heart

Table 2 represents the data recorded for the effects of new synthetic derivatives of thiazole acetic acid on the heart rate recorded as beats per minute in isolated rat hearts. The observations suggested that all the tested doses of the new derivatives reduced the heartbeat, but a significant reduction (*p* < 0.05) was only found for SMVA-60 when it was tested at 10 µM. The analysis to draw the significance of the difference was performed by comparing the data of the new synthetic derivatives with the data of the control. 

### 2.3. Effects of New Synthetic Derivatives of Thiazole Acetic Acid on Developed Tension in Isolated Rat Heart in Presence of Either Adrenaline or Acetylcholine

The effects of new derivatives of thiazole acetic acid in the presence of either adrenaline (Adr) or acetylcholine (Ach) were tested to record the influence of treatment on the developed tension in isolated hearts. The observations suggested that Adr at 6 µM significantly (*p* < 0.001) increased the developed tension compared to the control group. Furthermore, when 3 µM concentrations of different synthetic compounds were tested in the presence of Adr, a significant (*p* < 0.001) increase in developed tension was observed compared to the Adr group, except for the SMVA-10 derivative. This derivative did not produce significant alteration in the developed tension.

**Figure 1:**
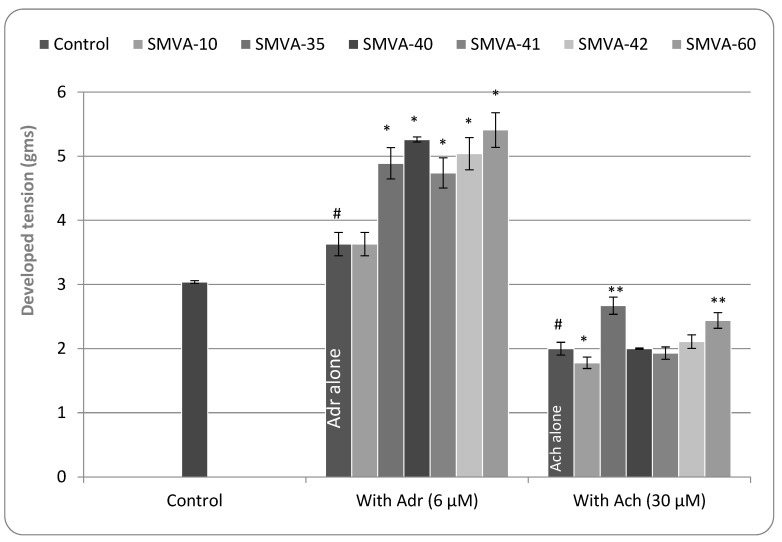
Effects of new synthetic derivatives of thiazole acetic acid on developed tension (gm) in isolated rat hearts in the presence of either adrenaline (Adr) or acetylcholine (Ach). Values are represented as mean ± SEM. Statistics: One-way ANOVA. # *p* < 0.001 compared with control and * *p* < 0.05, ** *p* < 0.01 compared with Adr/Ach.

Additionally, when Ach (30 µM) was tested alone, a significant (*p* < 0.001) reduction in developed tension was observed compared to control. In the presence of Ach, SMVA-10 induced a significant (*p* < 0.05) reduction in the developed tension, while SMVA-35 and SMVA-60 produced significant (*p* < 0.01) elevations in the developed tension compared to Ach (Figure 1).

### 2.4. Effects of New Synthetic Derivatives of Thiazole Acetic Acid on Heart Rate in Isolated Rat Heart in Presence of Either Adr or Ach

**Figure 2:**
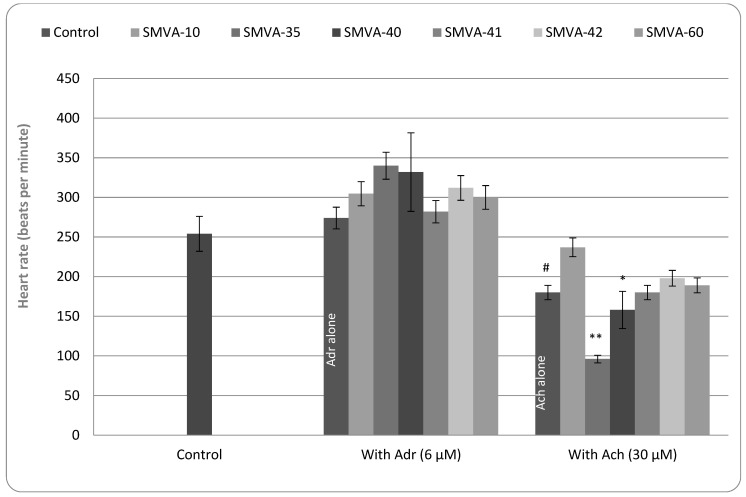
Effects of new synthetic derivatives of thiazole acetic acid on the heart rate (beats per minute) in isolated rat hearts in the presence of either Adr or Ach. Values are represented as mean ± SEM. Statistics: One-way ANOVA. # *p* < 0.05 compared with control and * *p* < 0.05, ** *p* < 0.001 compared with Ach.

The influence of Adr/Ach alone or when combined with the new synthetic derivatives of thiazole acetic acid on the heart rate is represented in Figure 2. When Adr (6 µM) alone was tested, no significant increase in the heart rate was observed compared to the control group. Additionally, when different derivatives of thiazole acetic acid at 3 µM were tested in the presence of Adr, an increase in the heart rate was observed but was found to be statistically non-significant when compared with Adr.

On the other hand, when Ach (30 µM) was tested alone, a significant (*p* < 0.05) reduction in the heart rate was observed compared to the control group. The addition of different synthetic compounds at 3 µM in combination with Ach (30 µM) indicated that SMVA-35 (*p* < 0.01) and SMVA-40 (*p* < 0.05) produced more reduction in heart rate compared to the Ach data. The other tested compounds induced variation, but this was found to be statistically non-significant (Figure 2).

### 2.5. Effects of New Synthetic Derivatives of Thiazole Acetic Acid on Contractile Response (%) in Isolated Blood Vessel and in the Presence of Prazosin (Praz)

**Figure 3:**
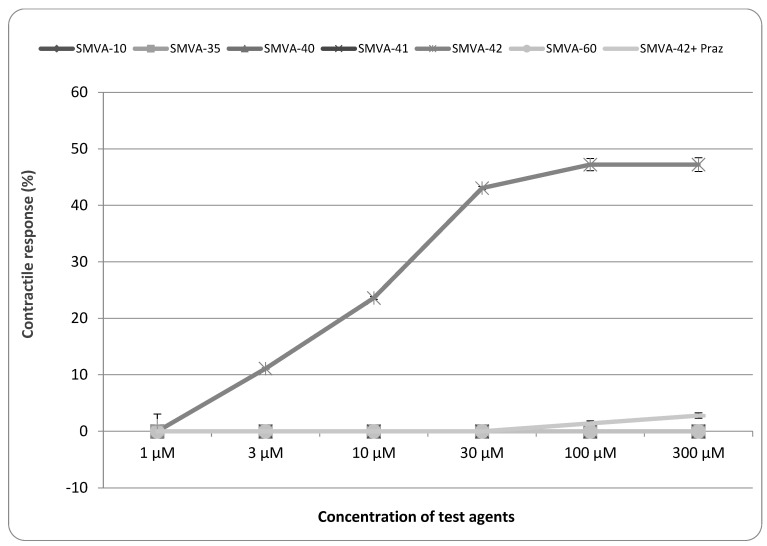
Effects of new synthetic derivatives of thiazole acetic acid on the contractile response (%) in isolated blood vessels and in the presence of Praz. Values are represented as mean ± SEM.

Different concentrations of new derivatives of thiazole acetic acid were tested to study their influence on the contractile response in the isolated blood vessel (aorta). The observations indicated that, except SMVA-42, none of the synthetic derivatives produced the contractile response in the isolated tissue. SMVA-42 produced a dose-dependent percentage increase in the contractile response; a peak/ceiling effect was observed at the 100 µM concentration. Praz, tested as a selective alpha-1 inhibitor, was found to reduce/abolish the effects of SMVA-42 on the isolated blood vessel. In the presence of Praz, only the higher doses, viz., 100 and 300 µM, produced the contractile response, to extents of 1.39% and 2.78%, respectively (Figure 3).

## 3. Discussion

The data from the present study suggest that most of the new synthetic derivatives of thiazole such as SMVA-35, SMVA-40, SMVA-41, and SMVA-42 significantly increased the developed tension in isolated hearts. The other two derivatives, viz., SMVA-10 and SMVA-60, enhanced the developed tension but only at lower doses. The developed tension, as reported in an earlier study, increases when the myocardial contraction is enhanced. It indicates the function of the work performed by the isolated heart [16].

For a better understanding, the new synthetic derivatives were tested in the presence of Adr. The observations indicated that Adr as well as the synthetic compounds (SMVA-35, SMVA-40, SMVA-41, SMVA-42 and SMVA-60) in combination with Adr significantly increased the developed tension (Figure 1). Adr, being a known agonist of the sympathetic system, binds to the beta-adrenergic receptors located in myocardial cells and activates the conversion of ATP to cAMP, which in turn activates the protein kinase. The activated protein kinase phosphorylates the voltage-sensitive calcium channels, opens them and allows more entry of calcium ions into the myocardium. The accumulated calcium then acts on the myofibrils, induces a sliding action, and produces ‘forceful’ contractions of the myocardium [16,17]. The greater increase in the developed tension when the derivatives of the thiazole were combined with Adr suggests that the combination potentiated the myocardial contraction [18].

In addition, when the derivatives such as SMVA-35 and SMVA-60 were tested in the presence of Ach, the reduction in the developed tension induced by Ach was found to be reversed, leading to the elevation of the developed tension. However, SMVA-10, in the presence of Ach, produced a greater reduction in the developed tension (Figure 1). The observations support the idea that some of the newer derivatives of thiazole acetic acid might have produced strong sympathetic actions, sufficient to overcome the cardiac-depressive effects of Ach [19]. A new synthetic derivative exhibiting potent action has also been reported in an earlier study. 2-Hydroxy-3-(phenoxypropyl) glycine is an aryloxy propranol amino acid derivative that has been shown to have a better cardiovascular effect than propranolol, which is considered a prototype beta-adrenergic blocker [20]. Furthermore, 2-(3-(9*H*-fluoren-9-ylideneaminooxy)-2-hydroxypropylamino)-3-methyl-butanoic acid, obtained from the *N*-alkylation of an amino acid with O-oxime ether, also produced a significant effect in reducing the heart rate, in addition to exhibiting considerable antibacterial activity [21].

The data from the present study also suggested that the new synthetic derivatives of thiazole non-significantly reduced the heart rate (Table 2). The observations indicate that, although the compounds possess the ability to increase the developed tension, the heart rate was not increased correspondingly. Such findings were seen in an earlier study too, where the test agent increased the developed tension, but the heart rate was found to be diminished [19]. The observation represents an interesting activity of the compounds. Such effects in increasing the developed tension by reducing the heart rate could be ‘beneficial’ in certain myocardial conditions such a failing heart [22]. The precise mechanism for this action could not be established from the present data; however, a suppressive effect on the pacemakers of the heart could be a possibility.

The study to determine the influence of known sympathomimetic/parasympathomimetic agents on the activity of newer derivatives of thiazole indicated that neither Adr nor the combination of synthetic compounds with Adr significantly altered the heart rate. However, Ach, when tested alone, significantly reduced the heart rate. SMVA-35 and SMVA-60 in the presence of Ach further suppressed the heart rate, suggesting that these compounds might augment the muscarinic action of Ach on the myocardium (Figure 2).

The effects of the new derivatives of thiazole acetic acid on the isolated blood vessels show that, with the only exception of SMVA-42, none of the compounds induced contractile responses on the tissue. The dose-dependent responses produced by SMVA-42 were found to be nearly abolished in the presence of Praz (Figure 3). The findings suggest that SMVA-42 might possess an agonistic effect towards the alpha-1 receptor, and this effect might complicate the pressure dynamics of blood vessels [23]. 

In the event of increased incidences of cardiovascular diseases around the world, the findings might provide an opportunity to test newer derivatives of thiazole that act mostly on the force of myocardial contraction [2]. However, more extensive studies are needed to establish the complete safety and efficacy of these synthetic compounds. 

## 4. Materials and Methods

### 4.1. Chemicals and Drugs

All the chemicals, reagents and solvents used in the synthesis of the new thiazole acetic acid derivatives were procured from the central house of the college, through the regular chemical supplier. The supplied agents were of analytical-grade quality, and all precautions were followed while handling, transporting, storing and utilizing the chemicals during the study. The sources of the drugs were as follows for adrenaline (Adnalin., Care Formulation Labs, Nasela, India), acetylcholine (Miochol, Bausch and Lomb, Anagni, Italy), prazosin (Prazopress, Sun Pharma Laboratories Ltd., Kamrup, India), ketamine (Anesketin, Northwich, UK), xylazine (Xylaealth, Livealth BioPharma Pvt Ltd., Mumbai, India) and heparin (Heparin injection, Flagship Biotech Int Pvt Ltd., Thane, India).

### 4.2. Synthesis of Thiazole Acetic Acid Derivatives

**Scheme 1:**
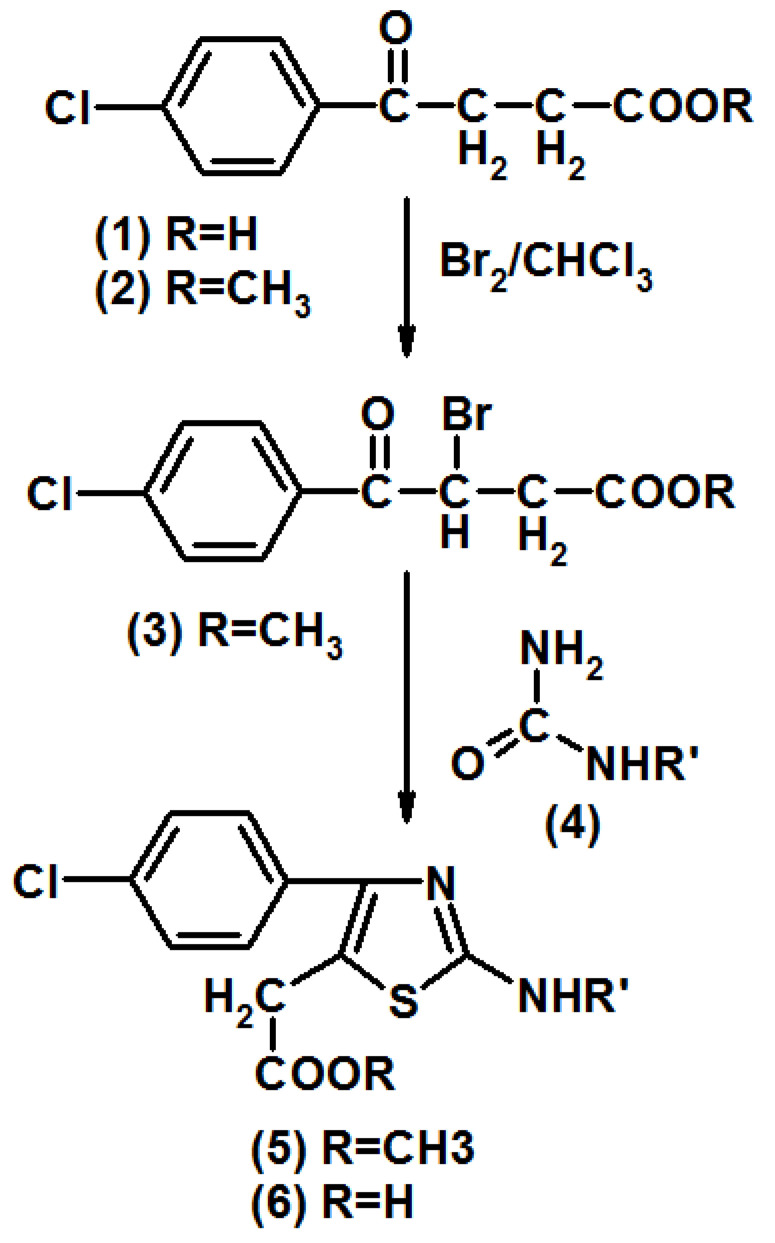
Scheme of the synthesis of the derivatives of thiazole.

The Friedel–Crafts acetylation method was used for synthesizing the newer derivatives of thiazole (Scheme 1), and the compounds having promising structure–activity relationships were selected for pharmacological screening [24]. The scheme of the synthesis, and the chemical and physical characteristics of the new synthesized compounds are provided in Table 3. In brief, the reaction was initiated by the acetylation of chlorobenzene with succinic anhydride in the presence of anhydrous aluminum chloride to give 3-(4-chlorobenzoyl) propionic acid (1). This compound was esterified in the presence of methanol, resulting in methyl-3-(4-chlorobenzoyl) propionate (2), and was further brominated to synthesize methyl-3-bromo 3-(4-chlorobenzoyl) propionate (3). The reaction was conducted in hot chloroform with continuous stirring.

The esterified bromine compound was then condensed with phenylthiourea and substituted phenyl thioureas in the presence of ethanol, with heating for 15 min. The substituted phenylureas (4) were prepared from different amines and ammonium thiocyanate with benzoyl chloride to obtain the substituted methyl-2-amino-4-(4-chlorophenyl) thiazole-5-acetate (5). Furthermore, saponification was performed on these compounds, resulting in the formation of new thiazole acetic acid derivatives (6). The purification and separation of the reaction products were conducted in the presence of 10% NaoH with reflexing for 2 h; then, the products were acidified and finally crystallized from ethanol. The compounds were filtered, dried and identified using IR spectra taken on a Phillips Pye Unicam SP-3200 IR spectrometer by using the KBr disc method. The proton nuclear magnetic resonance (NMR) spectra of the new synthesized compounds were obtained using an EM390 CW-NMR 90 MHz instrument, with CDCl_3_ as the solvent [24].

### 4.3. Experimental Animals

Laboratory-bred female Wistar albino rats weighing between 200 and 250 g were housed at 25 ± 5 °C in a well-ventilated animal house under a 12:12 h light and dark cycle. The rats had free access to standard rat chow (Amrut Laboratory Animal feed, Maharashtra, India) containing protein at 22.10%, oil at 4.13%, fiber at 3.15%, ash at 5.15% and sand (silica) at 1.12% (*w/w*) and water *ad libitum*. The animals were acclimatized in the laboratory conditions for one week prior to the start of the experiment. During this period, care was taken to monitor the health of the animals, and diseased animals (if any) were immediately returned to the central animal house for further care or treatment. The experiment was conducted after approval from the Institutional Animal Ethics Committee of Al-Ameen College of Pharmacy, Bangalore, India. The animals were maintained under standard conditions in an animal house approved by the Committee for the Purpose of Control and Supervision on Experiments on Animals (CPCSEA).

### 4.4. Treatment Protocol

Different concentrations of the six new derivatives of thiazole ranging from 1 nM to 300 µM were tested on the isolated hearts and blood vessels as per the procedures described in the literature. The derivatives of thiazole acetic acid were first tested alone to record their effects on isolated tissues and then in combination with a known agonist and antagonist. The known agents were acetylcholine (30 µM), adrenaline (6 µM) and prazosin (30 µM). Before the addition of a new drug or concentration, the isolated tissues were stabilized for 15–30 min in the presence of KH solution under standard-temperature conditions.

### 4.5. Experimental Procedure

The experimental animals were anaesthetized by administering a combination of ketamine hydrochloride (65 mg/kg i.p.) and xylazine (7.5 mg/kg i.p.). Heparin (100 units) was injected intraperitoneally 15 min prior to anesthesia. From the anesthetized rats, heart with at least 1 cm of attached aorta was removed as quickly as possible and placed in a porcelain dish containing KH solution at room temperature saturated with carbogen (95% O_2_ and 5% CO_2_), with occasional squeezing to remove the blood. The aorta was located and dissected free, and all the other vessels connected to the heart were trimmed away. The aorta was cut just below the point where it divided, and the heart was transferred to the perfusion apparatus; the aorta was then tied onto the cannula [25].

The heart was then perfused through the aorta (KH solution) with a flow rate of 5 mL/min at 37 °C by means of a peristaltic pump. A fine thread was tied to the apex of the heart and passed through a thermostatically controlled water-jacketed moist chamber and then through a pulley to the transducer. The resting tension and developed tension were recorded in the transducer. The resting tension (2 gm) is the basal tension required for recording the contraction of the heart. The developed tension is the tension in grams developed during the contraction at the optimum resting tension of the heart and is a function of the work performed by the isolated heart [26]. 

The isolated blood vessel experiment recorded the contractile or relaxant response, which resulted from the interaction of the agonist with the receptors. The isolated rat aortic blood vessels were mounted in a temperature-controlled tissue/organ bath and suspended in KH solution that was aerated with carbogen gas (95% O_2_ and 5% CO_2_) and connected to a transducer [27]. 

### 4.6. Statistical Analysis

The results are expressed as the mean ± SEM. The statistical significance was assessed using one-way analysis of variance (ANOVA) followed by Tukey’s multiple-comparison tests. *p* < 0.05 was considered significant.

## 5. Conclusions

The present study evaluated the cardiovascular effects of new synthetic derivatives of thiazole acetic acid. The findings suggested that most of the compounds possess the ability to increase the developed tension without affecting the heart rate and blood vessel activity. SMVA-10 (2-(phenyl amino)-4-(4-chlorophenyl) thiazole-5-acetatic acid), in the presence of Ach, reduced the developed tension, while SMVA-60 (ethyl 2-(phenyl amino)-4-(4-chlorophenyl) thiazole-5-acetate) increased it. Furthermore, both SMVA-35 (2-(2-chlorophenyl amino)-4-(4-chlorophenyl)thiazole-5-acetatic acid) and SMVA-40 (2-(4-methyl phenyl amino)-4-(4-chlorophenyl) thiazole-5-acetatic acid) were found to decrease the heart rate in the presence of Ach. SMVA-42 (2-(4-bromo phenyl amino)-4-(4-chlorophenyl) thiazole-5-acetatic acid) alone was found to produce the contractile response in the isolated aorta, and the effects were abolished when it was tested with prazosin. The agents might benefit patients diagnosed with failing hearts. However, elaborate research is essential to determine the exact role of these compounds, including toxicological studies, to ascertain their therapeutic potential.

## Figures and Tables

**Table 1:** Effects of new synthetic derivatives of thiazole acetic acid on the developed tension (gm) in isolated rat hearts.

Treatment	Concentration of New Derivatives of Thiazole Acetic Acid					
	1 nM	10 nM	100 nM	1 µM	10 µM	100 µM
Normal	2.05 ± 0.03					
SMVA 10	2.77 ± 0.24 *	2.57 ± 0.39	2.60 ± 0.30	2.70 ± 0.34	2.57 ± 0.54	2.31 ± 0.41
SMVA 35	2.68 ± 0.31	2.79 ± 0.27 *	2.79 ± 0.36 *	2.86 ± 0.42 *	2.68 ± 0.14	2.79 ± 0.39 *
SMVA 40	3.04 ± 0.19 **	2.98 ± 0.21 *	2.84 ± 0.41 *	2.81 ± 0.28 *	2.64 ± 0.29	2.72 ± 0.17 *
SMVA 41	3.11 ± 0.09 ***	3.00 ± 0.50 **	3.19 ± 0.22 ***	3.00 ± 0.15 **	3.26 ± 0.22 ***	3.26 ± 0.23 ***
SMVA 42	3.09 ± 0.24 **	3.09 ± 0.25 **	4.00 ± 0.08 ***	4.00 ± 0.46 ***	-	3.03 ± 0.32 **
SMVA 60	3.12 ± 0.29 ***	2.84 ± 0.18 *	2.93 ± 0.61 *	2.59 ± 0.23	2.22 ± 0.39	2.59 ± 0.20

Values are represented as mean ± SEM. Statistics: One-way ANOVA. * *p* < 0.05, ** *p* < 0.01, *** *p* < 0.001 compared with normal.

**Table 2:** Effects of new synthetic derivatives of thiazole acetic acid on the heart rate (beats per minute) in isolated rat hearts.

Treatment	Concentration of New Derivatives of Thiazole Acetic Acid					
	1 nM	10 nM	100 nM	1 µM	10 µM	100 µM
Normal	324.67 ± 8.12					
SMVA-10	236.00 ± 50.12	301.00 ± 33.52	254.00 ± 36.41	236.67 ± 31.96	249.00 ± 32.96	233.33 ± 30.65
SMVA-35	292.00 ± 41.23	290.00 ± 23.41	292.00 ± 28.64	286.67 ± 26.41	276.00 ± 19.65	263.33 ± 23.65
SMVA-40	276.00 ± 39.21	300.00 ± 51.97	280.67 ± 30.96	295.00 ± 19.98	290.00 ± 36.74	291.00 ± 19.98
SMVA-41	272.00 ± 26.34	259.50 ± 38.65	257.33 ± 40.31	254.00 ± 35.62	256.67 ± 23.95	239.00 ± 31.95
SMVA-42	243.33 ± 31.62	240.66 ± 20.96	252.00 ± 38.62	240.66 ± 29.64	-	254.66 ± 24.67
SMVA-60	242.67 ± 44.36	232.5 ± 36.41	242.33 ± 26.34	256.00 ± 24.65	220 ± 22.98 *	236.00 ± 26.14

Values are represented as mean ± SEM. Statistics: One-way ANOVA. * *p* < 0.05 compared with normal.

**Table 3:** Chemical and physical characteristics of the new derivatives of thiazole acetic acid.

New Synthetic Compound	R’	R	Melting Point	% Yield
SMVA-10	C_6_H_5_	H	240–242	80
SMVA-35	2-Cl C_6_H_4_	H	208–210	81
SMVA-40	4-CH_3_ C_6_H_4_	H	248–250	74
SMVA-41	4-F C_6_H_4_	H	261–262	81
SMVA-42	4-Br C_6_H_4_	H	238–240	80
SMVA-60	C_6_H_5_	C_2_H_5_	119–120	55
